# Cerebellar irradiation does not cause hyperactivity, fear, and anxiety-related disorders in the juvenile rat brain

**DOI:** 10.1186/s41747-022-00307-8

**Published:** 2022-11-15

**Authors:** Yafeng Wang, Cuicui Xie, Yiran Xu, Yaodong Zhang, Changlian Zhu, Kai Zhou

**Affiliations:** 1 Henan Neurodevelopment Engineering Research Center for Children, Zhengzhou Key Laboratory of Pediatric Neurobehavior, Children’s Hospital Affiliated to Zhengzhou University, Zhengzhou, China; 2 Department of Hematology and Oncology, Children’s Hospital Affiliated to Zhengzhou University, Henan, Children’s Hospital, Zhengzhou Children’s Hospital, Zhengzhou, China; 3 Centre for Brain Repair and Rehabilitation, Institute of Neuroscience and Physiology, University of Gothenburg, Gothenburg, Sweden; 4 Henan Key Laboratory of Child Brain Injury and Henan Pediatric Clinical Research Center, The Third Affiliated Hospital and Institute of Neuroscience, Zhengzhou University, Zhengzhou, China

**Keywords:** Anxiety, Cerebellum, Models (animal), Hyperactivity, Radiotherapy

## Abstract

**Background:**

The cerebellum is involved in hyperactivity, fear, and anxiety disorders that could be induced by whole-brain irradiation (WBI). However, whether cerebellar irradiation alone (CIA) could induce these disorders is unknown. We investigated the effect of CIA in an animal model.

**Methods:**

Eleven-day-old rat pups underwent a single 3-Gy dose of either WBI (*n* = 28) or CIA (*n* = 20), while 34 rat pups were sham-irradiated (controls). Cell death was evaluated in the subgranular zone of the hippocampus by counting pyknotic cells after haematoxylin/eosin staining at 6 h after irradiation for 10, 8, and 9 pups, respectively. Behavioural changes were evaluated via open-field test at 6 weeks for 18, 12, and 25 pups, respectively. Unpaired two-tailed *t*-test and one-way and two-way repeated ANOVA were used.

**Results:**

Massive cell death in cerebellar external granular layer was detected at 6 h after CIA (1,419 ± 211 mm, mean ± S.E.M. *versus* controls (68 ± 12 mm) (*p* < 0.001)), while no significant difference between CIA (1,419 ± 211 mm) *versus* WBI (1,433 ± 107 mm) (*p* = 0.955) was found. At open-field behavioural test, running distance, activity, wall distance, middle zone visit times, and duration were higher for WBI *versus* controls (*p* < 0.010), but no difference between CIA and controls was found (*p* > 0.05).

**Conclusions:**

Although the cerebellum is involved in hyperactivity, fear, and anxiety disorders, CIA did not induce these disorders, indicating that WBI-induced cerebellar injury does not directly cause these behavioural abnormalities after WBI. Thus, targeting the cerebellum alone may not be enough to rescue or reduce these behavioural abnormalities after WBI.

## Key points

- Cerebellar irradiation depleted external granular layer in the cerebellum.
- Cerebellar irradiation alone did not cause hyperactivity which could be induced after whole brain irradiation.
- Cerebellar irradiation alone did not cause fear and anxiety behavioural changes which could be induced after whole brain irradiation.

## Background

Posterior cerebellar tumours are the most common paediatric intracranial tumours; they include medulloblastomas, ependymomas, and pilocytic astrocytomas [1]. Radiotherapy is still one of the most efficient treatments for brain tumours; however, the concomitant side effects dramatically decrease the patient’s quality of life [1–5]. There has been an increasing number of survivors thanks to improved treatments, and this increase has accentuated concerns regarding the quality of life.

The cerebellum had been considered as solely contributing to motor functions. However, studies have revealed that the cerebellum plays an essential role in cognitive functions [6, 7]. Moreover, cerebellar irradiation can induce cognitive dysfunctions [8–10]. The cerebellum is also involved in fear and anxiety-related disorders, although the exact role that the cerebellum plays is still unknown [11, 12]. Several other brain regions, including the amygdala, prefrontal cortex, insula, and hippocampus, have been known to compose the anxiety circuitry [13–22].

Our previous study revealed that whole-brain irradiation (WBI) leads to hyperactivity, fear, and anxious behavioural changes in rodent models [23–25]; however, the mechanisms and which brain regions are involved are still unknown. We demonstrated that WBI could cause cerebellar volume reduction, precursor cell death, Purkinje cell disruption, inflammatory response, blood-brain barrier, and microvessel damage [26, 27]. Whether cerebellar irradiation alone (CIA) could lead to hyperactivity, fear, and anxiety-related disorders is unknown. A better understanding of the involvement of the irradiated cerebellum in behavioural anomalies is needed to understand the mechanisms by which radiotherapy induces behavioural changes and to develop novel therapeutic strategies.

We hypothesised that CIA causes hyperactivity, fear, and anxiety disorders. Thus, our aim was to evaluate these behavioural functions after CIA.

## Methods

### Animals and irradiation

Six-day-old male Wistar rat pups were purchased from Charles River Laboratories (Germany) together with the dam and housed in a pathogen-free and temperature- and humidity-controlled facility on a 12-h light/dark cycle and were provided free access to food and water. Each cage was included one mother and 10 pups. The pups from each litter were randomly grouped into three groups at postnatal day 11: sham-irradiated control group (*n* = 34), WBI group (*n* = 28), and CIA group (*n* = 20), for a total of 82 rat pups. The irradiation groups’ rats were anaesthetised with 50 mg/kg tribromoethanol (Avertin, Sigma-Aldrich, Stockholm, Sweden) and then placed in a prone position on a Styrofoam bed. The head was covered with a 1-cm tissue-equivalent bolus material after locating the whole brain with a 2 × 2 cm and cerebellum with a 0.5 × 2 cm radiation field, respectively. A single 3 Gy dose of radiation was delivered at a rate of 2.3 Gy/min by a linear X-ray accelerator (Varian Clinac 600CD, Radiation Oncology Systems, San Diego, CA, USA). The control group rat pups were also anaesthetised with 50 mg/kg tribromoethanol and placed in a prone position on a Styrofoam bed. They stayed the same amount of time in the radiation field as the irradiated rats but without initiating the radiator. This study was approved by the by the Gothenburg Animal Ethics Committee (202/2012), and all the experimental methods were performed in accordance with the guidelines for animal experiments of Gothenburg University.

Our previous experimental and clinical studies have shown sex difference in the cell death mechanisms and severity of brain injury in the immature brain after insult [28–31]. Whole brain irradiation in young rats have shown different impact on male and female [23, 32]. Furthermore, animal behaviour in females is much more influenced by the sexual cycle [33]. To avoid the potential influence of sex hormone on the behavioural test, we used male rat pups only in this study.

### Brain slice preparation and haematoxylin and eosin staining

The rats were deeply anaesthetised and perfused intracardially with phosphate-buffered saline and 5% formaldehyde in phosphate-buffered saline. Then, the brains were dissected and immersed in 5% formaldehyde at 4°C for 24 h. The tissue was embedded in paraffin after dehydration using a graded ethanol series and xylene. Next, the paraffin-embedded left hemispheres were cut into 5-μm sagittal sections, followed by haematoxylin and eosin staining [34]. Briefly, the wax was first removed, and the tissue rehydrated by incubating in xylene followed by a graded ethanol series. Then, a haematoxylin nuclear stain was applied, followed by eosin counterstaining after washing. Next, the sections were rinsed and dehydrated with a graded ethanol series and xylene. Finally, the sections were mounted by using Vector mounting medium.

### Cell number quantification and microscope

In the subgranular zone (SGZ) of the hippocampus, all pyknotic cells indicated by haematoxylin and eosin staining were counted. The length of the SGZ was measured to calculate the density of pyknotic cells. In the cerebellum, all pyknotic cells indicated by haematoxylin and eosin staining were counted on lobules 2−4 of same-layer sections. The interlayer length of the external granular layer (EGL) was measured to calculate the density of pyknotic cells. All counting was performed by a person blinded to the group information.

### Behavioural tests

The open-field test is sensitive to detect irradiation-induced behavioural abnormities in rats [23]. It was performed by using a 100 × 100 cm open-field arena. Movement was recorded automatically by the EthoVision 3.1 video-tracking software. The central zone was defined as a 30 × 30 cm area in the centre of the arena. The rats acclimatised to the open-field test room with water and food for 1 h, and then they were put in the open-field arena for a total of 20 min. The data were summarised and analysed for every 5-min interval.

### Statistics

All statistical analyses were performed on GraphPad, and in order to indicate the precision of the data represent the truth, all data are presented as the mean ± standard error of the mean. The unpaired two-tailed Student *t*-test and one-way ANOVA were used when comparing two groups and three groups, respectively. A two-way repeated ANOVA was used when comparing behavioural changes with time. Significance was considered when *p* was lower than 0.05.

## Results

### Cerebellar irradiation induced milder cell death in the hippocampus compared with WBI

**Fig. 1:**
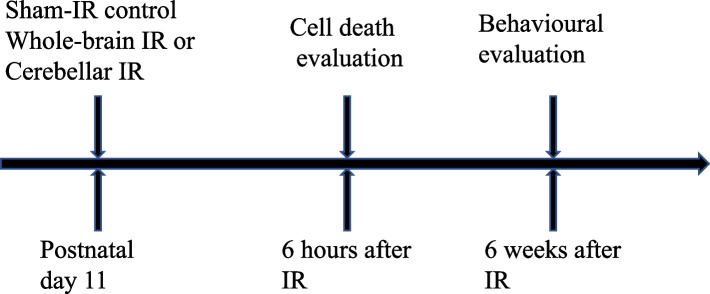
Schematic view of the study process. The pups were subjected to irradiation at postnatal day 11. A total of 27 pups (9 for control group, 10 for whole-brain irradiation (WBI) group, and 8 for cerebellar irradiation alone were killed at 6 hours for cell death evaluation. A total of 55 pups were used for behavioural evaluation at 6 weeks after irradiation. *IR*, Irradiation.

**Fig. 2:**
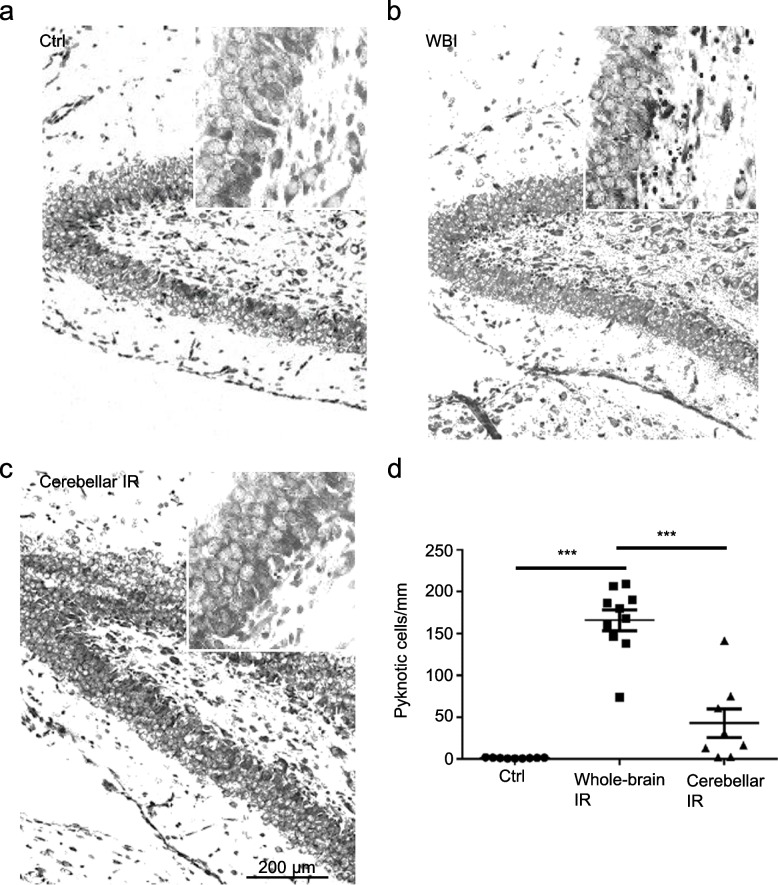
Cell death in the hippocampal subgranular zone (SGZ) after whole-brain irradiation (WBI) and cerebellar irradiation alone (CIA). Representative black-and-white images of haematoxylin and eosin staining showing the nuclear morphology in the hippocampal SGZ of a control rat (**a**), a rat subjected to WBI (**b**), and a rat subjected to CIA (**c**). **d** Graph showing the dead cell density in the control, WBI, and CIA groups; each dot represents one mouse. The data are presented as the mean ± standard error of the mean (*n* = 8−10). ***p < 0.001 by one-way Analysis of variance (ANOVA). *IR*, Irradiation.

To confirm the radiation delivered to the cerebellum is not leaked to the hippocampus that is sensitive to irradiation-induced cell death, pyknotic cells were counted in the SGZ in the dentate gyrus of the hippocampus to evaluate stem/precursor cell death 6 h after irradiation (Fig. 1). A massive number of dead cells were detected in the SGZ after WBI (*p* < 0.001, Tukey’s multiple comparisons test, one-way ANOVA). Some dead cells were also detected in the SGZ after cerebellar irradiation, but without significant difference compared to the control group (*p* = 0.062, Tukey’s multiple comparisons test, one-way ANOVA). Moreover, the number of dead cells in the SGZ after cerebellar irradiation was significantly less than after WBI (*p* < 0.001, Tukey’s multiple comparisons test, one-way ANOVA) (Fig. 2a–d).

### Both WBI and CIA induced the same level of cerebellar injury

**Fig. 3:**
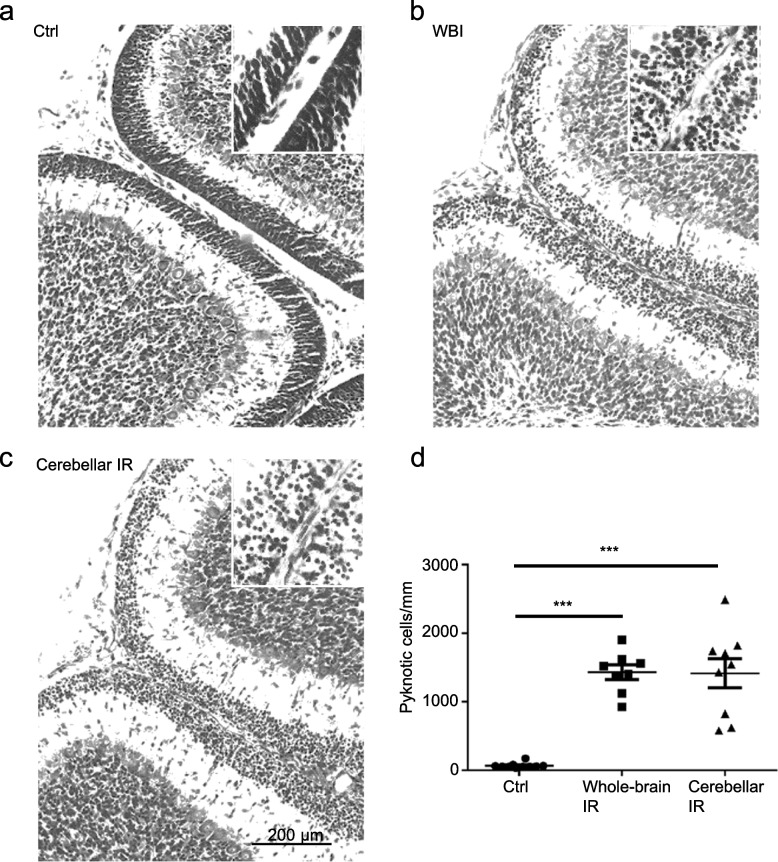
Cell death in the cerebellar external granular layer**.** Representative black-and-white images of haematoxylin and eosin staining showing the nuclear morphology in the cerebellum of a control rat (**a**), a rat subjected to whole-brain irradiation (WBI) (**b**), and a rat subjected to cerebellar irradiation alone (CIA) (**c**). **d** Graph showing the dead cell density in the control, WBI, and CIA groups; each dot represents one mouse. The data are presented as the mean ± standard error of the mean (*n* = 8−10). ***p < 0.001 by one-way ANOVA. *IR*, Irradiation.

The cerebellar injury was evaluated by counting the pyknotic cells in the EGL after irradiation (Fig. 1 and Fig. 3a–c). Both WBI and CIA induced massive cell death in the cerebellar EGL ((*p* < 0.001 for both), Tukey’s multiple comparisons test, one-way ANOVA), and there was no significant difference between these two groups in terms of dead cell numbers (*p* = 0.997, Tukey’s multiple comparisons test, one-way ANOVA) (Fig. 3a–d).

### CIA did not cause hyperactivity

**Fig. 4:**
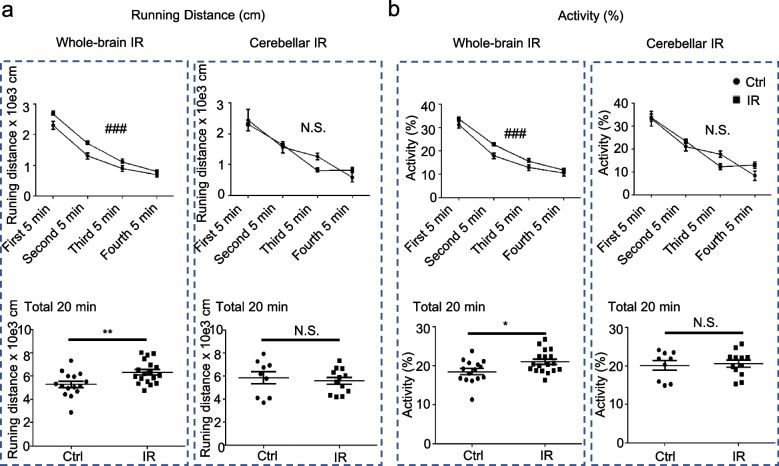
Assessment of activity in the open-field test. **a** The upper graphs show the running distance every 5 min for a total of 20 min. ^###^*p* < 0.001 by two-way repeated ANOVA comparing the control and whole-brain irradiation (WBI) groups (*n* = 16−18). N.S. stands for no significant difference by two-way ANOVA comparing the control and cerebellar irradiation groups (*n* = 9−12). The lower graphs show the distance run over 20 min. ** *p* < 0.01 by unpaired two-tailed Student *t*-test comparing the control and WBI groups (*n* = 16−18). N.S. stands for no significant difference by unpaired two-tailed Student *t*-test comparing the control and cerebellar irradiation alone (CIA) groups (*n* = 10−12). **b** The upper graph shows the activity every 5 min for a total of 20 min. ^###^*p* < 0.001 by two-way ANOVA comparing the control and WBI groups (*n* = 16–18). N.S. stands for no significant difference by two-way ANOVA comparing the control and CIA groups (*n* = 9−12). The lower graphs show the distance run over 20 min. *p < 0.05 by unpaired two-tailed Student *t*-test comparing the control and WBI groups (*n* = 16−18). N.S. stands for no significant difference by unpaired two-tailed Student *t*-test comparing the control and CIA groups (*n* = 10−12). For all graphs, the data are presented as the mean ± standard error of the mean. *IR*, Irradiation.

The open-field test was performed to evaluate the activity of 8-week-old male rats (6 weeks after irradiation) (Fig. 1). Over time, running distance and activity were reduced in the control, WBI, and CIA groups (*p* < 0.0001, two-way repeated ANOVA). In addition, running distance and activity were significantly higher in the WBI group than in the control group in each 5-minutes interval (*p* < 0.001 for both, two-way repeated ANOVA) and for a total of 20 minutes of free exploration (*p* = 0.006, and *p* = 0.020, respectively, unpaired two-tailed Student *t*-test). However, there was no difference between the CIA and the control groups (*p* = 0.637 or 5-min interval of running distance and *p* = 0.704 for 5-min interval of activity, two-way repeated ANOVA; *p* = 0.637 or 20 min of running distance, and *p* = 0.766 for 20 min of activity, unpaired two-tailed Student *t*-test) (Fig. 4a, b).

### CIA did not reduce anxious behaviour

**Fig. 5:**
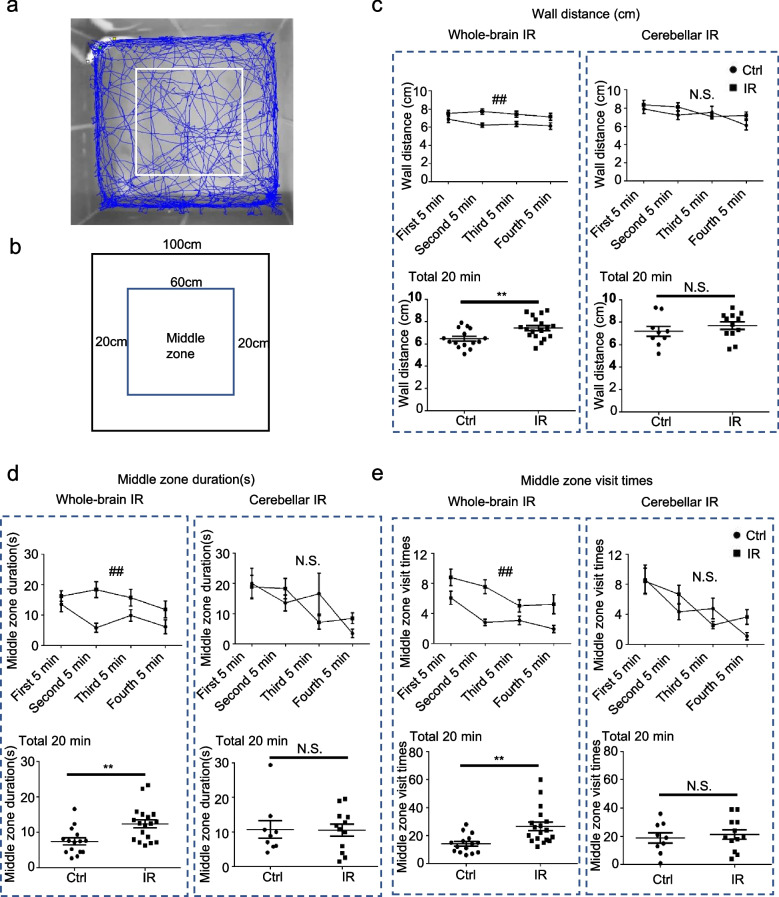
Assessment of fear and anxiety from the open-field test. **a** A picture showing the open-field arena. The blue line is the running trace of the rats, and the white rectangle is the middle zone. **b** A schematic showing the dimensions of the open-field arena. **c** The upper graphs show the distance from the wall every 5 min for a total of 20 min. ^##^*p* < 0.01 by two-way ANOVA comparing the control and whole-brain irradiation (WBI) groups (*n* = 16−18). N.S. stands for no significant difference by two-way ANOVA comparing the control and cerebellar irradiation alone (CIA) groups (*n* = 9−12). The lower graphs show the wall distance traveled over 20 min. ** *p*< 0.01 by unpaired two-tailed Student *t*-test comparing the control and WBI groups (*n* = 16−18). N.S. stands for no significant difference by unpaired two-tailed Student *t*-test between the control and CIA groups (*n* = 10−12). **d** The upper graphs show the time spent in the middle zone every 5 min for a total of 20 min. ^##^*p* < 0.01 by two-way repeated ANOVA comparing the control and WBI groups (*n* = 16−18). N.S. stands for no significant difference by two-way ANOVA comparing the control and CIA groups (*n* = 9−12). The lower graphs show the time spent in the middle zone over 20 min. **p < 0.01 by unpaired two-tailed Student’s *t*-test comparing the control and WBI groups (*n* = 16−18). N.S. stands for no significant difference by unpaired two-tailed Student *t*-test (*n* = 10−12). **e** The upper graphs show the number of times the middle zone was visited for every 5-min interval for a total of 20 min. ^##^*p* < 0.01 by two-way ANOVA comparing the control and WBI groups (*n* =16−18). N.S. stands for no significant difference by two-way ANOVA comparing the control and CIA groups (*n* = 9−12). The lower graphs show the number of visits to the middle zone over 20 min. **p < 0.01 by unpaired two-tailed Student *t*-test comparing the control and WBI groups (*n* = 16−18). N.S. stands for no significant difference by unpaired two-tailed Student *t*-test (*n* = 10−12). For all graphs, the data are presented as the mean ± standard error of the mean. *IR*, Irradiation.

Data from the open-field test (the distance from the wall, the time spent in the middle zone, and the number of visits to the middle zone) were used to evaluate fear and anxious behaviour (Fig. 5a, b). The WBI group has longer distance from the wall than control group in both analysis from 5-min interval (*p* = 0.002, two-way repeated ANOVA) and total 20 min of free exploration (*p* = 0.006, unpaired two-tailed Student *t*-test) (Fig. 5c). Moreover, WBI group spent more time in the middle zone (*p* = 0.001 for 5-min interval analysis, two-way repeated ANOVA; *p* = 0.003 for total 20 min free exploration, unpaired two-tailed Student *t*-test) and visited more times to the middle zone than the control group (*p* =0.001 for 5-min interval analysis, two-way repeated ANOVA; *p* = 0.002 for total 20 min free exploration, unpaired two-tailed Student *t*-test) (Fig. 5d, e). However, no difference of both visit duration and times was detected between the control and CIA groups in each 5-min interval (*p* = 0.956, and *p* = 0.631, respectively, two-way repeated ANOVA) and for the total 20 min of free exploration (*p* = 0.957, and *p* = 0.631, respectively, unpaired two-tailed Student *t*-test) (Fig. 5d, e).

## Discussion

Brain radiotherapy is still one of the most efficient ways to treat brain tumours. WBI is widely used to prevent cranial metastases, despite the accompanying behavioural deficits [35, 36]. For brain region-specific tumours, conformal strategies reduce the irradiation dose to the surrounding tissues, but behavioural deficits still occur [37, 38]. The mechanisms and brain regions involved in irradiation-induced behavioural deficits are largely unknown. The current study has shown that CIA does not cause hyperactivity, fear, and anxious behavioural abnormalities, all of which were detected for the WBI in the current study, as was in a previous study [23]. Thus, we conclude that CIA is not sufficient to cause abnormal activity, fear, and anxiety, despite the important role of the cerebellum in these functions and the severe injury due to cerebellar irradiation. This finding is important to understand the role of the cerebellum in irradiation-induced behavioural deficits, such as fear and anxiety disorders, which is a crucial issue in developing treatment strategies.

Neurons migrate from their proliferating site in the EGL to their final destination, the inner granular layer in the cerebellum; this migration is essential to forming synaptic connections and neuronal circuits [39–41]. The proliferation and migration are regulated by complex signals from other cells types [42, 43]. We have demonstrated that cerebellar irradiation leads to massive cell death in EGL, a finding consistent with our previous WBI study [26]. However, CIA did not induce hyperactivity, fear, and anxiety, all behaviours for which the cerebellum is involved. One possible explanation could be the cerebellar self-repair system. Nesting positive precursor cells in the Purkinje cell layer can switch the cell fate to generate granule cells to compensate for the cells lost after irradiation [44]. However, this repair mechanism is centrally regulated by Purkinje cells, which irradiation also affects [26]. Thus, irradiation not only depletes the EGL but may also injure the cerebellar self-repair system by disrupting Purkinje cell layers. Another possibility is that those different brain regions coordinate hyperactivity, fear, and anxiety disorders [45–47]; thus, CIA is insufficient to cause these disorders. Conversely, WBI could injure most brain regions; for example, it could kill a large number of neuronal precursors in the hippocampal SGZ, as found in the current study and in previous studies [23, 48]. Thus, CIA may not be enough to induce behavioural abnormalities such as anxiety and fear because it damages a much more restricted area of the brain.

The current study has some limitations. First, only male rats were used in the present study, both genders should be used to investigate sex differences in future studies. Second, only the open-field test was used to evaluate the behavioural abnormities; other tests, such as elevated-plus maze and fear conditioning, can be used to confirm the results in future studies. Third, clinical data should be combined with the preclinical model to further confirm the conclusion in future studies.

In conclusion, although the cerebellum is involved in different normal central nervous system functions and diseases such as hyperactivity, fear, and anxiety disorders, CIA does not cause these abnormalities, even though it causes a severe cerebellar injury, including massive cell death of EGL. This outcome is different compared with what occurs after WBI. Hence, targeting the cerebellum alone is not enough to rescue WBI-induced hyperactivity, fear, and anxiety disorders.

## Data Availability

The data supporting the findings are available upon request from the corresponding author.

## Abbreviations

CIA: Cerebellar irradiation alone
EGL: External granular layer
SGZ: Subgranular zone
WBI: Whole-brain irradiation
